# Heart Rate Variability as an Indicator of Chronic Stress Caused by Lameness in Dairy Cows

**DOI:** 10.1371/journal.pone.0134792

**Published:** 2015-08-13

**Authors:** Levente Kovács, Fruzsina Luca Kézér, Viktor Jurkovich, Margit Kulcsár-Huszenicza, János Tőzsér

**Affiliations:** 1 MTA–SZIE Large Animal Clinical Research Group, Üllő-Dóra Major, Hungary; 2 Institute of Animal Husbandry, Faculty of Agricultural and Environmental Science, Szent István University, Gödöllő, Hungary; 3 Department of Animal Hygiene, Herd Health and Veterinary Ethology, Faculty of Veterinary Science, Szent István University, Budapest, Hungary; 4 Department and Clinics of Reproduction, Faculty of Veterinary Science, Szent István University, Budapest, Hungary; ETH Zurich, SWITZERLAND

## Abstract

Most experimental studies on animal stress physiology have focused on acute stress, while chronic stress, which is also encountered in intensive dairy cattle farming–e.g. in case of lameness–, has received little attention. We investigated heart rate (HR) and heart rate variability (HRV) as indicators of the autonomic nervous system activity and fecal glucocorticoid concentrations as the indicator of the hypothalamic–pituitary–adrenal axis activity in lame (with locomotion scores 4 and 5; n = 51) and non-lame (with locomotion scores 1 and 2; n = 52) Holstein-Friesian cows. Data recorded during the periods of undisturbed lying–representing baseline cardiac activity–were involved in the analysis. Besides linear analysis methods of the cardiac inter-beat interval (time-domain geometric, frequency domain and Poincaré analyses) non-linear HRV parameters were also evaluated. With the exception of standard deviation 1 (SD1), all HRV indices were affected by lameness. Heart rate was lower in lame cows than in non-lame ones. Vagal tone parameters were higher in lame cows than in non-lame animals, while indices of the sympathovagal balance reflected on a decreased sympathetic activity in lame cows. All geometric and non-linear HRV measures were lower in lame cows compared to non-lame ones suggesting that chronic stress influenced linear and non-linear characteristics of cardiac function. Lameness had no effect on fecal glucocorticoid concentrations. Our results demonstrate that HRV analysis is a reliable method in the assessment of chronic stress, however, it requires further studies to fully understand the elevated parasympathetic and decreased sympathetic tone in lame animals.

## Introduction

Lameness is the number one welfare issue in the dairy industry due to pain, suffering and economic impact [1]. Recent studies found that in Hungarian dairies, on average 27–35% [2,3]—with extremes of 65% [4]—of lactating cows are clinically lame. Most of the physiological studies on dairy cattle welfare have focused on acute stress, while chronic stress, which has a more pronounced effect on welfare and production, has received little attention.

According to recent research on humans, geometric [5] and non-linear [6,7] measures of heart rate variability (HRV), i.e. the short-term fluctuations in successive cardiac interbeat intervals (IBI), are suitable for chronic stress assessment. Non-linear techniques are derived from chaos theory and non-linear system theory [8] and are effective to describe processes in biological systems [9]. The non-invasive measurement of heart rate and HRV has increasingly been used for the assessment of stress in dairy cattle [10].

Traditional methods for the evaluation of the cardiac IBI in time- and frequency-domains are extensively studied both in humans and animals. According to several papers on HRV analysis in farm animals certain cardiac parameters provide a powerful means for measuring the sympathetic and the parasympathetic activity of the autonomic nervous system (ANS) separately [11–13]. The behavioral and physiological responsiveness of dairy cows and calves exposed to short-term stressors e.g. machine milking, veterinary procedures, disbudding or castration has been extensively studied using ANS-related linear HRV indices (see Ref. [10] for a review). The majority of these studies found traditional HRV measures useful for the detection and evaluation of acute stress. Although non-linear HRV and its relation with chronic effects of different milking systems have been studied in dairy cows [14], to our knowledge, no research has been published on the effects of chronic stress caused by multifactorial disorders such as lameness using geometric or non-linear HRV in adult cattle.

It is widely accepted that measuring cortisol concentrations is useful to detect the effects of stress in animal species [15]. The small number of works on cattle in response to chronic stress has mainly evaluated cortisol in blood. Studies considering serum cortisol concentrations has been published in lame cows, with contradictory findings [16,17]. A direct relationship between fecal glucocorticoid metabolites, blood cortisol, and adrenal activity has been demonstrated in dairy cattle [18]. Due to the intestinal passage time [19] the metabolites of cortisol appear in feces 12–24 hours after cortisol has been excreted into blood and thus has proven to be useful for the evaluation of chronic stress [15,20].

Fecal cortisol metabolites have not been used in studies on stress caused by lameness in cattle so far. The aim of our study was to test whether concentrations of fecal glucocorticoid metabolites and/or HRV parameters are suitable to detect chronic stress conditions caused by lameness in dairy cows. We investigated non-linear characteristics of HRV as well as parameters from the time, frequency and geometric domain.

## Materials and Methods

### Animals and housing

This study was carried out on a commercial dairy farm in Hungary, with Holstein-Friesian cows. The herd had year-round calving patterns with annual rolling and an average herd size of 1200 cows and a 305-day milk yield of an average 8,840 kg/cow. This study was approved by the Department of Epidemiology and Animal Protection of the Directorate of Food Chain Safety and Animal Health at Central Agricultural Office (Permit Number: 22.1/1266/3/2010). All procedures involving animals were approved by the Ethics Committee of the Faculty of Veterinary Science, Szent István University.

The study was conducted during a 3-week period in November 2012. Hundred-and-forty-three animals were selected from the herd for this study. The farm was visited 3 to 4 times per week, and 8–10 animals were examined on each day. All selected cows were inspected physically before the HR recording. Animals with health problems or with a recent history (less than one month) of clinical mastitis or postpartum uterine diseases (n = 11) were excluded from the experiment to help exclude other possible causes of stress of pathological origin. Three of the cows were also excluded due to technical problems (dried out electrodes, being disturbed by group mates during data recording). Temperamental animals (n = 2) and cows in oestrus (n = 4) were not involved. Four cows were excluded due to equipment failure which prevented further analysis of HRV. Locomotion scorings were carried out twice. The first session was conducted 2 weeks before measurement and the second at the time of actual measurements on a 5-point scale according to Sprecher et al. [21] to ensure the scores had not drifted over time.

Locomotion scorings were carried out as cows exited the milking parlor and returned to the feeding bunk. Cows were observed walking on flat, nonslip concrete in a well-lit location [22]. The third author did the scoring, who was trained to use this locomotion scoring system with on-farm experience. Only those animals were involved in the lame group, which had a locomotion score 4 or 5 two weeks before HRV recordings. Animals with locomotion score 3 (n = 16), were not involved in the experiment. Finally, 51 lame (without any pharmacological treatment, with visible lesions on at least one hoof, with locomotion scores 4 and 5) and 52 non-lame (clinically healthy, without any pharmacological treatment, with locomotion scores 1 and 2) cows were included in the study (Table 1).

**Table 1:** Production data of experimental animals used for this study (Mean ± SD).

Health category	Age (years)	Parity	Days in milk	Daily milk yield (kg)	Body condition score^a^
Non-lame (n = 52)	3.8 ± 1.1	2.3 ± 0.4	143.8 ± 10.0	34.3 ± 7.5	2.6 ± 0.2
Lame (n = 51)	4.6 ± 1.3	2.5 ± 0.8	142.4 ± 9.2	32.2 ± 7.6	2.2 ± 0.2

^a^ranging from 1 = very lean to 5 = fat.

Cows were housed in modern free-stall barns bedded with straw. They were fed ad libitum with TMR consisting of 31.4% corn silage, 21.6% grass silage, 11.5% alfalfa hay, and 35.5% concentrate mash on a dry matter basis. Cows had free access to water. Animals were milked twice daily (between 5:00 am and 8:00 am and between 5:00 pm and 8:00 pm) in a 2 × 24 parallel milking parlor (BouMatic, Madison, WI, USA).

### HR and HRV

Heart rate recordings were obtained using a mobile recording system which included a Polar Equine T56H transmitter with two integrated electrodes, a Polar H2 heart rate sensor and a Polar RS800 CX heart rate monitor (Polar Electro Oy, Kempele, Finland). Electrode sites were covered with ample ultrasound transmission gel (Aquaultra Blue, MedGel Medical, Barcelona, Spain). Transmitters and electrodes were positioned as advised by von Borell et al. [13] and fitted to cows with a leather girth after morning milking (between 6:00 am and 8:30 am). Data recording started 2 hours after preparation and lasted until the evening milking has finished (between 6:00 pm and 8:30 pm).

As advised in earlier reports [14,23,24] IBIs recorded during undisturbed lying bouts were involved in the analysis. Focal cows were not removed from their production groups and, consequently, from their familiar surroundings. Lying periods were recorded by visual observations, from a distance of at least 6 m from the resting area. Observers used watches synchronized with the heart rate receivers to later match behavior with the individual’s heart rate recordings.

For analysis, equal lengths of 5-min time windows were selected from the IBI signal stream [13,25]. A total of 1084 valid 5-min time windows were used for HRV analysis, 560 from lame cows [Means ± SD (range); 10.0 ± 1.3 (8–11) per cow] and 524 from sound ones [Means ± SD (range); 10.3 ± 1.5 (9–12) per cow]. The Kubios HRV software (version 2.1, Biomedical Signal Analysis Group, Department of Applied Physics, University of Kuopio, Finland) was used for HRV analysis [26]. For artefact correction, the custom filter was used and set at 0.3, identifying IBIs differing from the previous IBI by more than 30% as artefacts. After abnormal interval removal, the algorithm of the program substitutes detected errors with interpolated intervals calculated from the differences between previous and next accepted IBIs. In addition, a visual inspection of the corrected data was performed to edit out any artefacts still existing (generally Type 4 and Type 5 errors, for details see Ref. [12]). For removing slow nonstationary trend components, the ‘smoothness priors’ based detrending approach was chosen with λ = 1000 and f_c_ = 0.029 Hz as used by a recent study on dairy cattle HRV [27].

The root mean square of successive differences (RMSSD) between consecutive IBIs were evaluated in the time domain, which is useful for detecting tendencies related to vagal tone in dairy cattle [10] and was used in our previous studies as well [28,29]. For computing frequency-domain HRV, IBI data were subjected to Fast Fourier Transformation (FFT) for power spectrum analysis [30]. The HRV spectrum was calculated with the FFT based Welch’s periodogram method set to 256s overlapping segments with 50% window overlap. The interpolation rate of IBI series was 4 Hz. Spectral parameters included the normalized power of the high-frequency (HF) band and the relative power of the low frequency (LF) component and HF (LF/HF). The HF component is strongly associated with vagal tone [31] while LF/HF gives information about the sympathovagal balance of the ANS [25,32]. The limits of the spectral components were set as follows: LF: 0.05–0.20 Hz, and HF: 0.20–0.58 Hz as advised by von Borell et al. [13].

For graphical representation of the correlation between successive IBIs, where each interval in the time series (IBI_i+1_) is plotted against its successor (IBI_i_), standard deviation 1 (SD1) and the ratio between standard deviation 2 (SD2) and SD1 (SD2/SD1) were calculated by Poincaré plot analysis. SD1 represents vagal nerve activity [33], while SD2/SD1 reflects the sympathovagal balance. Both parameters have been used in dairy cattle [28,34–36].

For geometric means of HRV the triangular interpolation of the IBIs (triangular interpolation of normal to normal, TINN) and the triangular index (RRtri index) were calculated. TINN approximates the IBI distribution by a linear function and the baseline width of this approximation triangle is used as a measure of the HRV [37,38]. The RRtri index is a measure, where the length of IBIs serves as the *X*-axis of the plot and the number of each IBI length serves as the *Y*-axis. The length of the base of the triangle is used and approximated by the main peak of the IBI frequency distribution diagram. This parameter is insensitive to artefacts and ectopic beats, because they are left outside the triangle [25].

For the calculation of non-linear parameters, the Recurrence Quantification Analysis (RQA) was used [39]. This procedure was already applied successfully in human medicine [40] and in animal models [41]. The L_MAX_ and the Shannon Entropy indices were chosen from RQA measures previously used in dwarf goat [24] and in dairy cattle [42].

L_MAX_ is the length of the longest line of recurrence points in a continuous row within the recurrence plot resulted from the RQA where a small L_MAX_ means a large amount of ‘chaos’ [13]. Shannon Entropy means the Shannon information entropy of the line length distribution and measures the complexity of the signal.

In addition, we used the Detrended Fluctuation Analysis (DFA), which was introduced by Peng et al. [43]. DFA is useful for analyzing time series that appear to be long-memory processes for determining the statistical self-affinity of a signal by quantifying the fractal scaling properties of short-interval IBI data series. In this study we evaluated short-term (DFA1, computed for 3–11 IBIs) and long-term fluctuations (DFA2, computed for 12–20 IBIs). These parameters reflect the amount of randomness in the IBI time series [8]. Besides RQA and DFA measures the Correlation Dimension Analysis (CDA) parameter Correlation Dimension (CD) was calculated. The CD value will be high for the chaotic data and it decreases as the variation of the IBI signal becomes less or rhythmic [11] therefore CD was found to be useful in determining pathological signals [9]. For the more detailed description of the mathematical background of non-linear characteristics of HRV calculated with the RQA, DFA and CDA methods see the work of Acharya et al. [11].

### Fecal cortisol metabolites

Since the concentration of cortisol metabolites in a fecal sample reflects the cortisol production after 12 hours in ruminants [15], feces samples for analysis of cortisol metabolites were collected two times daily for the evaluation of the activity of the hypothalamic-pituitary-adrenal (HPA) axis. About 50 to 100 g of feces was obtained manually from the rectum in the morning (immediately after fixing the heart rate monitors, between 6:00 am and 8:30 am) and in the evening (before removing the heart rate monitors, between 6:00 pm and 8:30 pm). Disposable gloves were changed after every cow. According to Palme [44], 10 to 15 g of feces from different locations on the glove was filled into sample tubes. Samples were stored on ice immediately and frozen at −20°C within 2 hours after collection until analysis as described by Möstl et al. [45]. For the extraction of the fecal glucocorticoid metabolites [46], samples were thawed at room temperature, stirred, and 0.5 g of feces was dispersed in 5 ml of 80% methanol [47]. The dispersion was vortexed for 30 min and centrifuged on +4°C at 3000 × g for 20 min [45]. The supernatant was transferred into Eppendorf tubes of 1.5 ml and stored at −20°C until further analysis. A H3 homemade radioimmunoassay was carried out to determine the concentration of fecal glucocorticoid metabolites following the description of Csernus [48]. All samples were analyzed in duplicate. Intraassay and interassay coefficients of variation were calculated.

### Statistical analysis

Statistical evaluation was carried out in SPSS 18 (SPSS Inc., Chicago, IL). The GLM univariate procedure was used for modeling the linear relationship between dependent scale variables (HRV indices and fecal cortisol concentrations—one model for each), and one or more categorical and scale predictors. Averaged values of all HRV parameters calculated for 5-min IBI segments were used for each animal in the statistical evaluation to avoid pseudo-replication. Categorical predictors (main effects) were selected as fixed factors (parity: 1, 2, 3, 4 lactations, days in milk: between 140 and 160 days, body condition score: 2, 2.5, 3, 3.5 and lameness category: non-lame and lame) in the models. Age of cows and milk yield on experimental days were included in the models as covariates. Before each analysis, Levene’s test was used for testing equality of error variances. Tests of between-subjects effects were also determined for all sources of variances. The confidence intervals were adjusted with the post hoc test by Bonferroni. The level of significance was set at 0.05.

## Results

None of the main factors had a significant effect on mean fecal glucocorticoid concentrations of samples collected either in the morning or in the evening. Fecal cortisol concentrations of lame and non-lame cows are shown in Table 2.

**Table 2:** Fecal cortisol concentrations of lame (n = 51) and non-lame (n = 52) dairy cows.

Health category	Fecal glucocorticoid concentrations (ng/g)	
	Morning	Evening
Non-lame	54.5 ± 8.6	57.2 ± 11.8
Lame	40.4 ± 8.7	44.6 ± 12.1
*P*-value	0.300	0.180
*F*_1,87_	1.09	1.83

Descriptive statistics are based on Mean ± SD of non-transformed values of fecal cortisol concentrations. *F*-values are the results of the GLM univariate procedure for each variable. *P*-values for differences between groups are based on results of the Bonferroni post hoc test.

Days in milk and body condition had no effect on HRV parameters. Parity had a slight effect on RMSSD (univariate test: *F*_3,87_ = 3.18, *P* = 0.007) and on L_MAX_ (univariate test: *F*_3,87_ = 9.60, *P* = 0.003). L_MAX_ was lower in first lactation heifers than in fourth lactation cows (157 ± 20 vs. 265 ± 32 ms, *P* = 0.025). In second lactation cows lower RMSSD was found (15.2 ± 2.5 ms) than in third or fourth lactation cows (19.8 ± 3.0 ms and 35.5 ± 5.7 ms, *P* = 0.045 and *P* = 0.028, respectively).

Lameness had a pronounced effect on the baseline cardiac activity of cows. Heart rate was approximately 10 beats/min lower in lame cows than in non-lame animals. Parasympathetic measures both in the time- (RMSSD) and frequency-domain (HF) were higher in lame cows than in non-lame ones, while the indices of sympathovagal balance (LF/HF and SD2/SD1) were lower in lame cows than in sound cows. All geometric and non-linear HRV measures were lower in lame cows compared to non-lame ones. Lameness had no effect only on SD1 parameter. Values of HRV parameters in lame and non-lame cows are presented in Table 3.

**Table 3:** Heart rate and heart rate variability parameters of lame (n = 51) and non-lame (n = 52) dairy cows during lying posture.

Cardiac parameter	Health category		Statistics	
	Non-lame	Lame	*P*-value	*F*_1,87_
Time-domain measures				
Heart rate (min^-1^)	77.7 ± 1.7	66.7 ± 1.8	<0.001	63.96
RMSSD (ms)	20.0 ± 3.3	25.2 ± 3.4	0.048	4.00
Geometric measures				
RRtri index (ms)	6.8 ± 0.6	5.3 ± 0.6	0.028	4.14
TINN	223.4 ± 20.8	137.3 ± 21.3	0.008	7.35
Frequency-domain measures				
HF (n.u.)	32.2 ± 3.6	63.1 ± 3.7	<0.001	119.91
LF/HF	2.2 ± 0.5	0.6 ± 0.3	<0.001	15.64
Poincaré measures				
SD1 (ms)	14.2 ± 2.4	17.4 ± 2.4	0.079	3.15
SD2/SD1	5.1 ± 0.6	3.1 ± 0.6	<0.001	19.59
Non-linear measures				
L_MAX_ (beats)	278.5 ± 23.7	221.2 ± 24.2	0.003	9.60
Shannon entropy	4.0 ± 0.1	3.4 ± 0.1	0.003	9.39
DFA1	1.24 ± 0.05	1.11 ± 0.06	0.010	7.03
DFA2	1.34 ± 0.07	1.05 ± 0.07	<0.001	27.15
CD	0.65 ± 0.06	0.49 ± 0.07	0.020	3.72

Descriptive statistics are based on Mean ± SD of non-transformed values of cardiac parameters. *F*-values are the results of the GLM univariate procedure for each variable. *P*-values for differences between groups are based on results of the Bonferroni post hoc test.

RMSSD: root mean square of successive R–R interval differences; TINN: triangular interpolation of normal to normal; RRtri index: IBI triangular index; the overall variability in R–R intervals; HF: normalised power of the high-frequency band; LF/HF: the ratio between the low-frequency (LF) and the HF band; SD1: standard deviation of instantaneous R–R variability measured from axis 1 in the Poincaré plot; SD2/SD1: the ratio between SD2 (standard deviation of long-term continuous R–R variability measured from axis 2 in the Poincaré plot) and SD1; L_MAX_: the longest diagonal line segment in a continuous row within the Recurrence Plot; Shannon entropy: the deterministic line segment length distributed in a histogram of the Recurrence Plot; DFA1: the short-term fluctuations in HRV (3–11 IBIs); DFA2: the long-term fluctuations in HRV (12–20 IBIs); CD: Correlation Dimension.

## Discussion

We investigated time-domain, frequency-domain, geometric and non-linear HRV parameters as well as fecal glucocorticoid concentrations for the description of chronic changes in the cardiac IBI time series and HPA axis activity in dairy cattle. Although a variety of techniques based on non-linear theories has been proposed to quantify HRV, there are only a few number of studies on the evaluation of non-linear HRV in dairy cattle. According to recent research on HRV analysis methods, non-linear indices have many advantages compared to traditional linear parameters: 1) higher insensitivity to the presence of error IBIs [11]; 2) better reproducibility [49]; 3) higher sensitivity to smaller modulations in HR [50]. Since our paper is the first that describe the effects of chronic stress and HRV in adult cattle, it is difficult to compare our results to earlier findings in this field. Most of the studies on the effects of chronic physiological load on HRV has involved humans and lab animal species.

With the exception of SD1, all cardiac variables were affected by lameness. Pairwise comparisons of the values of linear indices highlighted marked differences in HR and ANS indices of HRV (HF and LF/HF) between lame and non-lame animals. Although heart rates of the lame cows were significantly lower than that of healthy cows, they were on average 18 beats/min higher than the threshold for pathological bradycardia (48 beats/min) [51]. Inconsistent with our results, earlier studies on conscious rats found that heart rate of animals exposed to prolonged intermittent stress [52] or chronic social stress [53] were similar to that of control animals. A recent human study on the cardiovascular consequences of chronic pain [7], found no differences in heart rate between affected and control patients. In line with our findings, Austin et al. [54] have reported on bradycardia in BSE positive cattle. The authors explain that though the disease affects the central nervous system primarily, including sites influencing heart rate, lower heart rates in infected animals is associated with low food intake. As lame cows tend to be spending more time lying and less time feeding [55], their lower heart rates may be associated with a low overall activity and food intake in our study as well. Since we found higher RMSSD and HF in lame animals compared to sound ones, it is possible that lameness-related bradycardia is mediated by increased vagal tone. Despite the conflicting reports on the functional roles of endogenous opioids in stress regulation [56], depressed heart rates of lame cows is possibly a result of the effect of endogenous opioids [57–60]. Some studies have reported on opioid secretion in a prolonged pain situation [61,62] and others highlighted the adaptation of opioid systems to protect the animals from excessive physiological reactions to stress [63]. A limitation of our study, that opioid parameters were not measured.

We found higher parasympathetic and lower sympathetic activity in lame cows (higher RMSSD, HF and lower LF/HF and SD2/SD1 ratios), while earlier studies reported on the predominance of the vagal tone indicated by a significant increase of RMSSD [64] or HF [65] in cows positive for BSE. Both results reflect on elevated parasympathetic tone in case of chronic physiological loads, however, it is not clear whether this phenomenon and a lower HR is a direct consequence of chronic stress or associated with a lower locomotor activity of lame animals. According to Nanda et al. [66], the HPA axis appears to be under suppressive opioidergic control, however, cattle under long-term pain may react in an opposite manner. The limtation of our study is that we did not determine locomotor activity or lying time and frequency of focal animals. In a recent work we found that lying time and lying frequency did not influence ANS activity in healthy cows (unpublished results), however, in this paper it cannot serve as a basis of interpretation regarding HR and vagal tone. For exact conclusions on lameness-related ANS activity further investigations are required where the behavior of animals as well as their overall activity is controlled.

Geometric measures of HRV (RRtri index, TINN) were lower in lame cows than in sound animals. These indices describe the overall variability in IBIs [25] and a reduction in these parameters is indicative of stress [67]. Our observations in lame cows are consistent with similar data obtained in patients with chronic emotional distress [68,69], chronic obesity [70], chronic cardiovascular [71] and pulmonary diseases [5], observing lower values of RRtri index and TINN compared to control subjects.

It is well known from studies in human cardiology that the time course of HRV contains non-linear chaotic components [72,73] and it is generally recognized that non-linear techniques are effective when describing biological systems [9]. Non-linear parameters of HRV have been studied in relation with chronic alterations in cardiac activity examining periods of undisturbed lying and thus of minimal physical activity for the analysis of the cardiac IBI [23,24]. The indices of non-linear HRV in the present study referred to a loss of general freedom in cardiovascular dynamics. Both RQA and DFA methods showed a difference between groups. All non-linear parameters calculated with RQA (L_MAX_, Shannon entropy) or with DFA (DFA1, DFA2) as well as Correlation Dimension were statistically lower in lame cows than in non-lame cows. The decreased value of complexity measures (L_MAX_, Shannon entropy) in lame cows reflects a change towards a more periodic heart rate under stress, indicating stronger regularity and decoupling of multimodal integrated networks within the cardiovascular system. This reduction in heart rate complexity in affected animals may reflect a lower adaptability and fitness of the cardiac pacemaker [14].

According to human studies, health state and well-being [6,74], or the function of the ANS [75,76] can be described by means of DFA indices. Krstacic et al. [77] observed a decrease in DFA parameters parallel with a decreased SNS activity in men with cardiomyopathy. This is in accordance with our findings on lower LF/HF and SD2/SD1 ratios in lame cows than in non-lame ones and a reduction in DFA1 and DFA2. In line with our results, Correlation Dimension was lower in patients with cardiac disorders [78] or with chronic mental stress disorder [79].

In this work, besides lameness, we involved production variables in the analysis to evaluate their possible effects on HRV. Days in milk and body condition score did not affect HRV parameters. Parity was the only factor that influenced cardiac activity but only in case of two parameters. RMSSD and L_MAX_ were higher in cows with higher parity. These results seem to be indicative for a lower vagal tone and a lower degree of complexity in cardiac dynamics in younger animals, however, the differences are so minor that no serious effect of parity on cardiac function can be testified.

Measuring fecal cortisol metabolites as a feedback-free method is referred to as a valuable tool in several research fields, e.g. animal welfare [15]. In case of monitoring prolonged periods of elevated glucocorticoid production, the precise sampling protocol is not that critical [80] and the concentrations of cortisol metabolites in a fecal samples reflect the cortisol production after 10–12 hours in cattle [15], we decided to collect feces samples two times daily. This regime enabled the undisturbed IBI recording for the entire experimental days. None of the production variables affected mean fecal glucocorticoid concentrations in the present study. In opposite to our expectations, we did not find a significant effect of lameness on fecal glucocorticoid concentrations either in samples collected in the morning, or in the evening. Walker et al. [17] also found similar cortisol concentrations in lame and sound cows, albeit they measured milk for cortisol. In earlier studies cortisol determination for feces has been judged a crude and highly variable parameter for stress analysis [81] due to the high inter-animal variations [80]. We tried to overcome this problem by involving a large number of animals in this study, however, our results might mirror the combined effects of several housing-associated stressors which could have masked the effects of lameness on the HPA activity. Another explanation of our results might be that cortisol responses gradually diminish with repeated stressor exposure [82]. Supposedly, a longer-term monitoring of fecal cortisol metabolites or cortisol concentrations in hair [83] would be demonstrative of HPA axis activation due to long-term stress caused by lameness.

Based on our findings, linear and non-linear analysis of HRV are promising approaches for chronic stress detection. Further research might be beneficial in the evaluation of treatments or newly developed technologies targeted on the reduction of the effects of chronic stress in dairy housing systems.

## Conclusion

Chronic intermittent stress—e.g. lameness—induces distinct changes in linear and non-linear components of HRV. Heart rate was lower, while vagal tone was higher in lame animals. The decline of non-linear heartbeat dynamics indicate a loss of complexity in heart rate and a more deterministic control of HRV in reaction to chronic stress load. It requires further research to understand whether elevated parasympathetic tone and depressed HR are associated with lower locomotor activity or the expression of long-term stress caused by lameness. It appears that chronic pain does not necessarily lead to a change in fecal cortisol concentrations.
