# Correction: Heart Rate Variability as an Indicator of Chronic Stress Caused by Lameness in Dairy Cows

**DOI:** 10.1371/journal.pone.0146625

**Published:** 2016-01-04

**Authors:** Levente Kovács, Fruzsina Luca Kézér, Viktor Jurkovich, Margit Kulcsár-Huszenicza, János Tőzsér

In the Funding section, the grant number from the funder Research Centre of Excellence is listed incorrectly. The correct grant number is: 9878/2015/FEKUT project.
